# Supplementary material for: Attosecond interferometry with self-amplified spontaneous emission of a free-electron laser
Source: Nat Commun. 2017 May 30;8:15626. doi: 10.1038/ncomms15626 (PMC5459985; doi:10.1038/ncomms15626)
Supplement: Supplementary Information — Supplementary Figures, Supplementary Notes and Supplementary References. [file ncomms15626-s1.pdf]

Supplementary Material

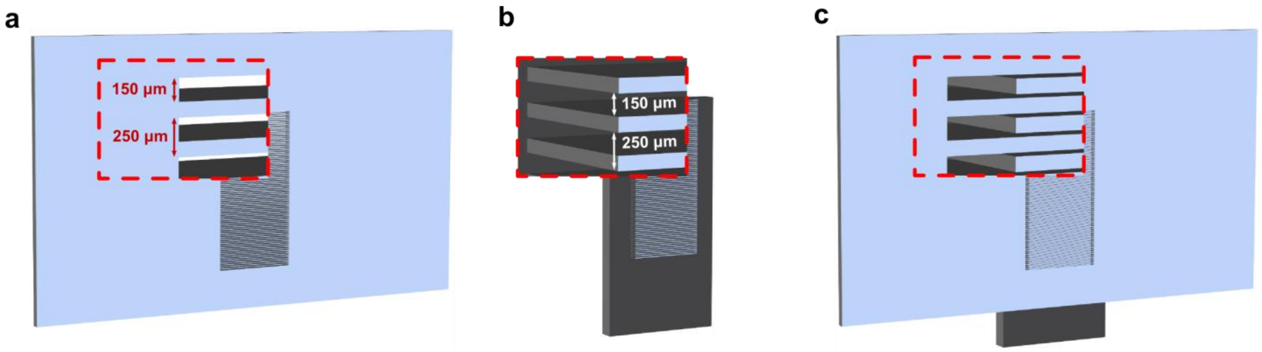

Supplementary Figure 1: Gratings of the split-and-delay unit. Two gratings of the split-and-delay unit (SDU) produced from silicon wafers: (**a**) slotted grid and (**b**) ridged grating. (**c**) Shows their arrangement when interleaved.

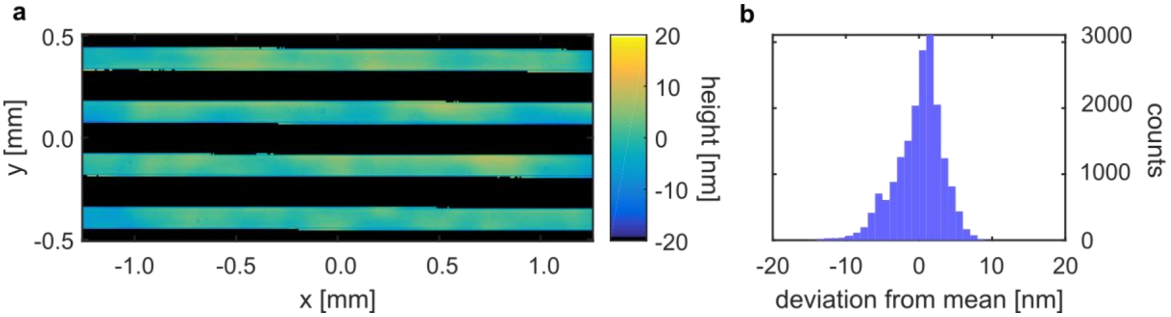

Supplementary Figure 2: Heightmap of the ridged grating. (**a**) The surface profile of the ridged grating obtained using the white light interferometer on the area illuminated by the free-electron laser (FEL) beam. (**b**) The histogram of (**a**).

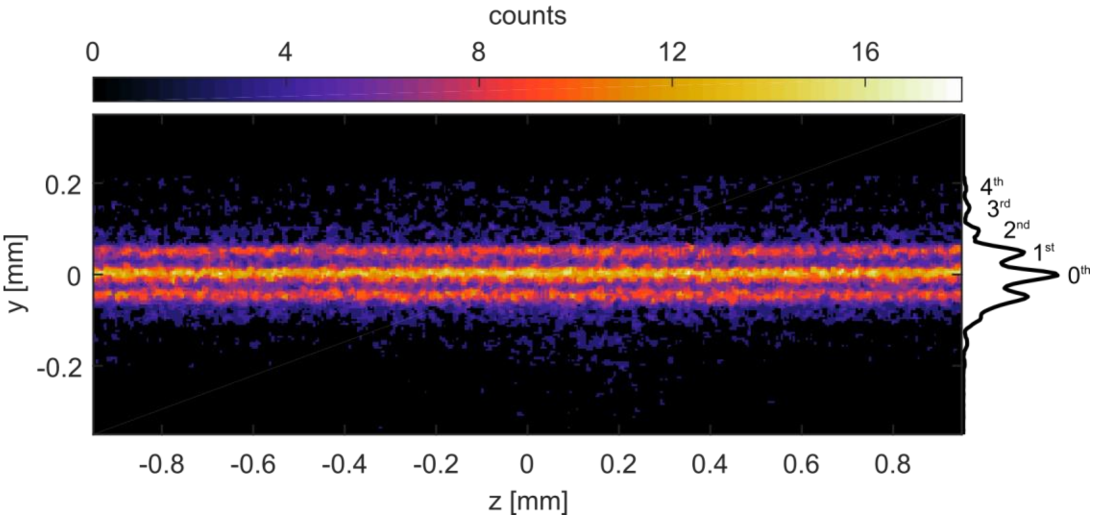

Supplementary Figure 3: Focused FEL beam after reflection from the ridged grating. The image was obtained by spatially-resolved detection of Xe^+ ions created by the 38 nm radiation of the free-electron laser (FEL) propagating along the *z*-direction. The image shows a number of diffraction orders separated by 46 µm. The integral of the image along *z* shown on the right side reveals orders up to 4th.

Supplementary Note 1: Possible application to shorter wavelengths

The scaling of the current setup to shorter wavelength faces some technological challenges as one is sensitive to the quality and position of the reflective surfaces on a sub-wavelength level (preferably better than $\lambda/4$). While controlling the average position of a surface with 1 nm precision is easily possible, vibrations on this scale have to be taken into account. While there is the possibility to reduce vibrations by careful damping of all relevant frequencies and using pumps without moving parts (ion getter pumps), we opted for measuring the instantaneous position of the gratings at the time of each FEL shot with a white light interferometer (WLI) as described above. With that, the question of how precisely one can control the position of the reflective surfaces and therefore the delay is transformed to how precisely one can measure it. With our WLI setup characterized to have a precision of about 3 nm in single-shot mode for the relative grating positions, this is perfectly fine for 38 nm (better than $\lambda/10$) and with some loss in contrast down to 12 nm wavelength ($\lambda/4$). As our characterization was mainly limited by the camera noise, there is the potential this can be extended down to the 1 nm level (the approximate resolution when operating the WLI in scanning mode). This would be good enough for sub-10 nm wavelengths interferometry. For even shorter wavelengths one would definitely need to reduce the incidence angle on the gratings in the setup (currently 22°) to maintain sufficient reflectivity. This helps the resolution as the actual optical path length difference (OPD) between the two copies of the FEL pulse is given by $2d \sin\alpha$, with α being the angle to the surface and d being the relative displacement of the surfaces. That means halving the incidence angle while also halving the wavelength, keeps the relative resolution of the OPD in units of the wavelength

constant. Taking this into account, the positional accuracy of the surfaces is likely not going to be an issue for employing this scheme to a few nanometers wavelengths. As shown in Supplementary Fig. 2 the planarity of the substrates with an RMS deviation of 3.2 nm is in the same range as the precision of our current WLI setup. Therefore, the same considerations apply. We expect to reach the 1 nm level by going to thicker substrates (less prone to warping due to internal stress) and optimized processing procedures.

Supplementary Note 2: Spatial imaging of ions

Images of the FEL spatial intensity distribution were obtained by detecting Xe^+ ions with an imaging TOF spectrometer. The system consists of a long (670 mm) Eppink-Parker velocity map imaging (VMI) spectrometer[1], a phosphor-based position-sensitive detector, and a camera. For the purposes of the experiment the spectrometer was operated in the spatial imaging mode. The spatial positions of the charged particles are resolved regardless of their velocities in this mode of operation as opposed to VMI. The spectrometer allows imaging the ionization volume with a magnification factor of 18. The fluorescence signal from the phosphor was observed with a CCD camera. The system enables spatial imaging of the ionization volume with a resolution of approximately 4.6 µm, which is sufficient to resolve the individual diffraction orders.

Supplementary Note 3: Monochromator

The details of the monochromator employed in the experiment are given elsewhere[2,3]. The slit width was set to 70 µm, which corresponds to a transmitted bandwidth of 0.005 nm at 38 nm wavelength. The reason for performing the study with a rather closed slit, i.e. narrow bandwidth, was solely given by the experimental task to resolve the individual diffraction orders induced by the SDU. Each order in the interaction region is an image of the exit slit of the monochromator, which limits us to small slit widths in the present geometry. Future experiments will make use of a toroidal focusing mirror instead of a spherical one, an ion microscope instead of a VMI, and an increased demagnification factor allowing for larger slits and therefore bandwidth. Ultimately, the use of a low bunch charge SASE operation mode results in only 1–2 longitudinal modes per FEL pulse on the 10 µJ level and a high degree of longitudinal coherence without the need for a monochromator.

Supplementary References

1. Eppink, A. T. J. B. & Parker, D. H. Velocity map imaging of ions and electrons using electrostatic lenses: application in photoelectron and photofragment ion imaging of molecular oxygen. *Rev. Sci. Instr.* **68,** 3477–3484 (1997).
2. Martins, M. et al. Monochromator beamline for FLASH. *Rev. Sci. Instr.* **77**, 115108 (2006).

3. Gerasimova, N., Dziarzhytski, S. & Feldhaus, J. The monochromator beamline at FLASH: performance, capabilities and upgrade plans. *J. Mod. Opt.* **58**, 1480–1485 (2011).
